# Comparative Analysis of Antibiotic Resistance in Acute Cholangitis Patients with Stent Placement and Sphincterotomy Interventions

**DOI:** 10.3390/life13112205

**Published:** 2023-11-13

**Authors:** Bogdan Miutescu, Deiana Vuletici, Calin Burciu, Felix Bende, Iulia Ratiu, Tudor Moga, Eyad Gadour, Felix Bratosin, Durganjali Tummala, Vasile Sandru, Gheorghe Balan, Alina Popescu

**Affiliations:** 1 Department of Gastroenterology and Hepatology, “Victor Babes” University of Medicine and Pharmacy Timisoara, Eftimie Murgu Square 2, 300041 Timisoara, Romania; miutescu.bogdan@umft.ro (B.M.); calin.burciu@umft.ro (C.B.); bendefelix@gmail.com (F.B.); ratiu_iulia@yahoo.com (I.R.); moga.tudor@yahoo.com (T.M.); popescu.alina@umft.ro (A.P.); 2 Advanced Regional Research Center in Gastroenterology and Hepatology, “Victor Babes” University of Medicine and Pharmacy Timisoara, 300041 Timisoara, Romania; 3 Department of Gastroenterology, Faculty of Medicine, Pharmacy and Dental Medicine, “Vasile Goldis” West University of Arad, 310414 Arad, Romania; 4 Department of Gastroenterology, King Abdulaziz Hospital-National Guard Health Affairs, Al Ahsa 31982, Saudi Arabia; eyadgadour@doctors.org.uk; 5 Department of Medicine, Zamzam University College, Khartoum 11113, Sudan; 6 Department of Infectious Diseases, “Victor Babes” University of Medicine and Pharmacy Timisoara, Eftimie Murgu Square 2, 300041 Timisoara, Romania; felix.bratosin@umft.ro; 7 Department of General Medicine, K.S. Hegde Medical Academy, Nityanandanagar, Deralakatte, Mangaluru, Karnataka 575018, India; anju9899@gmail.com; 8 Department of Gastroenterology, Clinical Emergency Hospital of Bucharest, 105402 Bucharest, Romania; drsandruvasile@gmail.com; 9 Department 5, “Carol Davila” University of Medicine and Pharmacy, 050474 Bucharest, Romania; 10 Department of Gastroenterology, “Grigore T. Popa” University of Medicine and Pharmacy, 700115 Iasi, Romania; balan.gheo@me.com

**Keywords:** acute cholangitis, antimicrobial resistance, bile duct obstruction

## Abstract

In response to rising concerns over multidrug resistance patterns in acute cholangitis patients, this retrospective study was conducted at the Emergency County Hospital Timisoara, Romania, encompassing patients treated between August 2020 and August 2023. The study aimed to investigate the influence of prior interventions, specifically sphincterotomy (with or without stent placement), on the current clinical and microbiological profiles of acute cholangitis patients. By differentiating between patients with a history of sphincterotomy and the endoscopic retrograde cholangiopancreatography (ERCP)-naïve, we assessed the resistance of bacterial strains to antibiotics by the Tokyo Guidelines 2018, using bile cultures from 488 patients. The study identified various multidrug-resistant organisms, with a total multidrug resistance incidence of 19.9%. Significant variations were observed in the distribution of specific microorganisms and resistance patterns across different intervention groups. Patients with previous interventions, particularly those with both sphincterotomy and stent, exhibited elevated white blood cells (WBC) and C-reactive protein (CRP) levels in comparison to their ERCP-naïve counterparts. This group also presented a striking prevalence of two bacteria in their bile cultures at 50.0%, compared to 16.1% in ERCP-naïve individuals. Regarding multidrug resistance, the prior sphincterotomy with stent placement had a prevalence of 50.0%. The presence of ESBL bacteria was also significantly higher in the same group at 28.7%, contrasting with the 8.9% in ERCP-naïve patients. Additionally, the same group had a higher burden of *Klebsiella* spp. infections, at 37.2%, and *Enterococcus* spp. at 43.6%. On the antibiotic resistance front, disparities persisted. Piperacillin/Tazobactam resistance was notably more rampant in patients with a previous sphincterotomy and stent, registering at 25.5% against 11.1% in the ERCP-naïve group. This study underscores a substantial discrepancy in multidrug resistance patterns and antibiotic resistance among acute cholangitis patients with previous manipulation of the bile ducts, without expressing significant differences by the type of stent used.

## 1. Introduction

Acute cholangitis (AC), a bacterial infection characterized by inflammation of the bile ducts, is a critical gastrointestinal condition with a noteworthy morbidity and mortality rate [1]. The mortality rate for acute cholangitis can vary based on a number of factors, including the severity of the condition, the timeliness of treatment, and underlying patient health factors, ranging between 1 and 10% with prompt treatment [2,3]. The higher severity end can typically reach mortality rates as high as 50%, being associated with multiple comorbidities or failure to perform biliary drainage [4,5,6,7]. Globally, acute cholangitis represents a major challenge, primarily precipitated by biliary obstruction from gallstones or malignancy. This condition necessitates a sizable number of emergency surgical interventions [8,9]. Distinct from primary biliary cholangitis, the global incidence of AC stands at approximately 8–12 cases per 100,000 individuals annually [10,11,12]. The wide range of AC patients encountered in healthcare systems underscores the importance of discerning the variables affecting disease progression and outcomes. With increasing cases, healthcare settings witness a diverse array of patients, which makes it essential to understand the differentiating factors that influence the disease progression and outcomes, namely, the underlying nature of the condition being malignant or benign, the intervention techniques employed, and existing comorbidities [13,14,15,16,17].

Increasing antibiotic resistance and the emergence of multidrug resistance patterns in bacterial pathogens that are responsible for different infections, including acute cholangitis, have exponentially escalated the complexity of managing this condition [18,19]. In the last decade, the alarming rise in antibiotic resistance has been corroborated by numerous studies, highlighting increased patient morbidity, prolonged hospital stays, and heightened financial strains on the healthcare system [20,21,22]. This crisis, further fueled by the inappropriate use of antimicrobial agents and natural selection pressure, calls for an in-depth investigation to streamline treatment protocols, particularly focusing on groups with varying underlying pathology and treatment interventions.

Stent placement and anterior sphincterotomy, routinely employed in the management of acute cholangitis, have demonstrated varying degrees of success and complications [23,24]. The utilization of these interventions, frequently determined by the malignancy status as a source of obstruction, has been noted to potentially influence the patient’s susceptibility to antibiotic resistance [25]. Consequently, analyzing the role these interventions play in dictating the cultures and antibiotic resistance patterns becomes pivotal.

Moreover, contemporary literature indicates a possible correlation between the nature of biliary obstruction (malignant vs. benign) and the bacterial flora present, which could subsequently influence antibiotic resistance patterns [26,27]. While malignancies involve complex pathological processes and potential immunosuppression, benign obstructions might present different bacterial profiles. By understanding these distinctions, we aim to provide a more comprehensive perspective on antibiotic resistance in acute cholangitis patients, irrespective of the underlying etiology [28].

In light of escalating antibiotic resistance concerns, there is a pressing need for a study offering a current perspective and establishing a foundation for ensuing research. Hence, this study endeavors to discern potential variances in multidrug resistance patterns among patients subjected to diverse intervention techniques. The study’s primary objectives encompass detailing culture and resistance outcomes among AC patients post sphincterotomy or stent placement; and meticulously examining antibiotic resistance patterns, spotlighting multidrug resistance variances as influenced by the stent material. 

## 2. Materials and Methods

### 2.1. Research Framework and Ethical Considerations

An analytical retrospective study was conducted at the Emergency County Hospital Timisoara, a prominent tertiary healthcare facility situated in western Romania. The study encompassed all individuals who underwent endoscopic retrograde cholangiopancreatography (ERCP) for biliary drainage owing to acute cholangitis (AC) from August 2020 to August 2023. Each patient had samples collected for bile and blood cultures. Essential medical details and personal data were extracted from the hospital’s medical archives and individual patient paper records. The resistance exhibited by bacterial strains to the antibiotics suggested by the Tokyo Guidelines 2018 (TG18) [29] was meticulously assessed. This research adhered to the ethical principles outlined in the 1975 Declaration of Helsinki and received approval from the internal review board.

### 2.2. Participant Selection and Sample Collection

AC was diagnosed following the criteria delineated in TG18. Participants were incorporated into the study once, during their initial admission, even though several had experienced multiple AC episodes throughout the data gathering timeframe. Exclusion criteria encompassed patients with AC that followed ERCP, and patients who were on antibiotic treatment for other conditions at the time of AC diagnosis.

Post admission, all participants were administered antibiotics, chosen based on the respective TG18 classifications [29], and subsequent establishment of AC diagnosis. The respective gastroenterology department involved in the study predominantly employed antibiotic regimens of ampicillin/sulbactam, ciprofloxacin, or levofloxacin for mild AC; ceftriaxone, cefepime, or piperacillin-tazobactam for moderate AC; and either meropenem or imipenem for severe cases. Pathogens in bile and blood samples were cultured and identified using appropriate mediums. Blood cultures were initiated upon admission for moderate and severe AC patients, in line with TG18 suggestions. Bile specimens were acquired post cannulation through the sphincterotome, preceding the therapeutic process. Initially, a minimum of 5 mL of the procured bile was discarded, followed by the collection of another 5 mL in a sterile vessel equipped with a medium conducive for anaerobic and aerobic bacterial cultures. The samples underwent a minimum of a seven-day incubation period at 37 °C until signs of microbial proliferation were evident. Antibiotic susceptibility assessments (minimum inhibitory concentration—MIC) were executed utilizing the VITEK^®^ 2 apparatus (bioMérieux, Marcy-l′Étoile, France), and the outcomes were analyzed based on the prevailing guidelines [30]. The susceptibility to antibiotics was defined according to the Clinical and Laboratory Standards Institute (CLSI) norms and criteria for all cultured bacteria [31].

Upon arrival, B-mode sonography was utilized to ascertain the underlying cause of obstruction. In scenarios where the diagnosis remained elusive, additional techniques like endoscopic ultrasound (EUS), contrast-enhanced CT, or CE-MRI were employed, which also aid in staging malignancies. Further diagnostic confirmation was achieved through the analysis of tumor markers and scrutinizing the histopathological data obtained from ERCP or EUS biopsies. ERCP was exclusively utilized as a therapeutic modality, facilitated by a therapeutic duodenoscope (Olympus Corp., Tokyo, Japan), with the common bile duct being accessed using a guidewire. ERCP interventions were carried out under sedation, administered by an expert anesthesia and intensive care team, using a combination of midazolam, propofol, and fentanyl, based on their established internal protocols. The timing for ERCP was dictated by disease severity and the stipulations of the Tokyo Guidelines, as determined by the endoscopists. In patients diagnosed with choledocholithiasis, the primary objective of ERCP was stone extraction. Complex cases of choledocholithiasis, where stone extraction posed challenges, warranted the placement of plastic stents. Depending on the individual diagnoses, either plastic or metal stents were employed, or no stents, depending on patients’ scenarios. 

### 2.3. Data Acquisition and Study Variables

Initially, patients were managed in the hospital based on the etiology of bile obstruction, classified as malignant or benign etiology. Further, the microbial identification and antibiotic resistance patterns were studied based on the procedure performed, splitting the study groups into patients with prior sphincterotomy, patients with previous sphincterotomy and stent placement, and those with no previous intervention, also known as ERCP-naïve [32]. During the study, a considerable array of variables was documented to facilitate an exhaustive analysis. This included the patients’ demographics (gender and age) and clinical manifestations, including abdominal pain, jaundice, fever, and chills. A laboratory analysis depicted a range of variables including white blood cell count (WBC), designated in the range of 4.0–9.5 × 10^3^/microliters, C-reactive protein levels (CRP), total bilirubin levels within a range of 0.2–1.2 mg/dL, platelet count indexed between 150 and 400 × 10^3^/microliters (PLT), and the international normalized ratio (INR).

The interventions and procedural data were recorded using variables such as previous sphincterotomy, previous stent placement, which was further detailed as metallic or plastic, the time until ERCP was performed, as well as the patient’s history of cholecystectomy. The clinical evaluations and outcomes were scrutinized through variables like blood culture and detected germs, the number of days hospitalized, and cases of weekend admissions. A pivotal aspect was the diagnostic categorization as malignant or benign and the underlying cause of obstruction, reported alongside the Tokyo severity score, categorized as I, II, or III.

Furthermore, an investigation into antibiotic resistance patterns was represented through variables such as multi-drug resistance (MDR), extended-spectrum beta-lactamases (ESBL), methicillin-resistant Staphylococcus aureus (MRSA), vancomycin-resistant enterococci (VRE), and carbapenem-resistant Enterobacteriaceae (CRE). The study meticulously noted the germ species identified and a comprehensive detailing of the antibiotic treatment administered, incorporating a wide spectrum of antibiotics such as ampicillin/sulbactam, piperacillin/tazobactam, ciprofloxacin, levofloxacin, cefepime, ceftriaxone, ceftazidime, cefuroxime, meropenem, imipenem, gentamicin, amikacin, ticarcillin/clavulanic acid, and trimethoprim-sulfamethoxazole. These antibiotics were scrutinized by hospital laboratory protocols and are advocated by the Tokyo Guidelines 2018 for treating AC, pending validation through bile or blood culture analysis.

### 2.4. Statistical Analysis

Data management and analysis were conducted utilizing the statistical software SPSS version 26.0 (SPSS Inc., Chicago, IL, USA). Continuous variables were represented as mean ± standard deviation (SD), while categorical variables were expressed in terms of frequencies and percentages. To analyze the changes between two means of continuous variables, the ANOVA test was performed. The Kruskal–Wallis test was used to compare median values between the three study groups. The chi-square test was utilized for the categorical variables. A *p*-value threshold of less than 0.05 was set for statistical significance. All results were double-checked to ensure accuracy and reliability.

## 3. Results

From our patient cohort of 488 individuals diagnosed with acute cholangitis, we observed a mean age of 69.3 years (SD ± 10.9). Males constituted 46.5% (n = 227) of the cohort. Broken down by age categories, the majority of our patients were older adults (>65 years), comprising 67.4% (n = 329) of the total. Middle-aged adults (40–65 years) represented 28.3% (n = 138), while young adults (18–39 years) were the least prevalent, making up only 4.3% (n = 21). In terms of clinical presentation, the most common symptom was jaundice, experienced by 89.5% (n = 437) of the patients. Abdominal pain was also a frequent complaint, reported by 73.3% (n = 358). Fever and chills were noted in 31.5% (n = 154) of the individuals.

Concerning the timing of ERCP intervention, 65.1% (n = 318) underwent the procedure emergently within 48 h, while 14.9% (n = 73) received it urgently between 48 and 72 h. A total of 19.9% (n = 97) had the procedure performed later than 72 h post presentation. The median duration of hospitalization for these patients was 8.6 days, with an interquartile range (IQR) of 5.3 days. Hospital admissions during weekends accounted for 27.0% (n = 132) of the cases. In our cohort, 20.9% (n = 102) underwent cholecystectomy. As per the Tokyo severity score, a significant majority had a Grade III severity at 83.6% (n = 408), followed by Grade I at 10.5% (n = 51), and Grade II at 5.9% (n = 29).

Regarding prior interventions, 73.8% (n = 361) were ERCP-naïve. A total of 6.7% (n = 33) had a previous sphincterotomy, while 19.3% (n = 94) had undergone both a sphincterotomy and stent placement previously. Lastly, when examining the etiology of the obstruction, the causes were nearly evenly split between malignant (50.4%, n = 246) and benign (49.6%, n = 242) factors, as detailed in Table 1.

The laboratory data were compared across the three distinct patient groups: those who previously underwent sphincterotomy (n = 33), those with both sphincterotomy and stent (n = 94), and the ERCP-naïve group (n = 361). For white blood cell (WBC) counts, patients with both a sphincterotomy and stent exhibited the highest mean value at 12.8 thousands/mm^3^, followed closely by the sphincterotomy-only group at 11.5 thousands/mm^3^. The ERCP-naïve group had a slightly lower mean count of 10.6 thousands/mm^3^. The observed differences were statistically significant, with a *p*-value of 0.003.

Levels of C-reactive protein (CRP) were also disparate. The group with both sphincterotomy and stent manifested the highest mean CRP levels at 116.9 mg/L. This was contrasted by the ERCP-naïve patients whose mean CRP was at 91.5 mg/L, while the sphincterotomy-only group hovered in-between at 108.3 mg/L. These variations were substantiated, with a *p*-value of 0.014. Total bilirubin levels seemed relatively consistent across all groups, with the differences not reaching statistical significance (*p*-value of 0.383). Platelet (PLT) counts were lowest in the ERCP-naïve group at a mean of 241.8 thousands/mm^3^, compared to the sphincterotomy-only group at 272.5 thousands/mm^3^, and 266.1 thousands/mm^3^ for those with both interventions. This discrepancy was statistically significant, with a *p*-value of 0.041.

A pronounced difference was observed in the international normalized ratio (INR). The ERCP-naïve group had a considerably lower mean INR value of 1.3, in stark contrast to the substantially elevated values of 3.0 and 3.3 in the sphincterotomy-only and sphincterotomy with stent groups, respectively. The divergence here was starkly significant, with a *p*-value of <0.001.

Differences in bacterial presence in bile cultures across groups were discernible. The ERCP-naïve patients had a significantly higher incidence of sterile bile (38.2%) compared to the other groups, while those with both a sphincterotomy and stent exhibited a pronounced presence of two bacteria (50.0%). Blood cultures displayed a similar trend; a significant proportion of ERCP-naïve patients had sterile blood results (74.8%), while the incidence of two bacteria was more pronounced in the group with both interventions at 11.3%, a significant difference with a *p*-value of 0.001, as presented in Table 2.

Table 3 describes the interventions performed based on the etiology of obstruction. Regarding benign etiologies in a total of 242 patients, choledocholithiasis was the predominant cause and showed a statistically significant difference across the groups. A total of 66.7% of the patients with a previous sphincterotomy, 5.0% of those with both a sphincterotomy and stent placement, and 29.8% of the ERCP-naïve patients had choledocholithiasis, with a *p*-value of <0.001. This indicates that the presence of choledocholithiasis varied significantly between these intervention groups. Other benign etiologies such as vaterian ampulloma, benign choledochal stenosis, Mirizzi syndrome, and liver abscess showed no significant differences across the groups, with *p*-values of 0.491, 0.342, 0.239, and 0.411, respectively.

For malignant etiologies, which included 246 patients, there were statistically significant variations in the prevalence of pancreatic cancer, cholangiocarcinoma, and malignant vaterian ampulloma across the groups. Pancreatic cancer was present in 15.2% of patients with a previous sphincterotomy, 41.5% of those with a sphincterotomy and stent, and 30.7% of ERCP-naïve patients, with a significant *p*-value of 0.002. Cholangiocarcinoma was diagnosed in 15.2% of the previous sphincterotomy group, 9.3% of the sphincterotomy and stent group, and 6.1% of the ERCP-naïve group, with a highly significant *p*-value of <0.001. Malignant vaterian ampulloma was noted in 0.0% of patients with only a previous sphincterotomy, 16.0% of those with both interventions, and 4.2% of ERCP-naïve patients, with another significant *p*-value of <0.001. Other malignancies like malignant extrinsic compression and gallbladder cancer did not differ significantly across the groups, with *p*-values of 0.501 and 0.588, respectively.

Across the 488 bile samples analyzed, the study discerned a range of multidrug-resistant microorganisms, with a total incidence of multidrug resistance (MDR) of 19.9%. ESBL was found in 12.3% of the total samples, illustrating notable variations among the groups (*p*-value < 0.001). Of significant mention, the group that underwent both sphincterotomy and stent placement displayed a pronouncedly elevated prevalence of ESBL at 28.7%. In contrast, the ERCP-naïve group registered an incidence of 8.9%, with the lowest prevalence of 3.0% observed in the sphincterotomy-only cohort.

With MRSA, its overall prevalence was relatively low at 0.4%. Both the combined sphincterotomy and stent group and the ERCP-naïve group showed minimal detection of MRSA at 1.1% and 0.3%, respectively. Remarkably, the sole sphincterotomy group recorded no instances of MRSA. VRE was discerned in 2.7% of the bile samples. The group with both sphincterotomy and stent placement revealed a higher incidence of VRE at 8.5%, in comparison to the ERCP-naïve group and sphincterotomy-only group at 1.1% and 3.0%, respectively, with a significant *p*-value of <0.001.

CRE was identified in 4.5% of the total samples. The group with both sphincterotomy and stent insertion recorded a relatively higher incidence at 11.7%, contrasting with the 2.8% in the ERCP-naïve group and 3.0% in the sphincterotomy-only group. The *p*-value was notably significant at <0.001. In the context of the overall MDR organisms, there was a marked difference between the groups, manifested by a *p*-value of <0.001. The group with both sphincterotomy and stent placement demonstrated a considerably elevated MDR prevalence at 50.0%. This was distinctively higher than the 13.0% in the ERCP-naïve cohort and the 9.1% in the group with only a sphincterotomy, as described in Table 4.

Table 5 delineates a comprehensive assessment of microbial characteristics identified in bile samples that were stratified based on distinct therapeutic strategies encompassing previous sphincterotomy, previous sphincterotomy accompanied by prior stent placement, and ERCP-naïve patients. The analysis revealed significant discrepancies in the distribution of specific microorganisms across various groups. Predominantly, *Escherichia coli* dominated with a prevalence of 30.7% across all samples. A conspicuous difference was noted across the cohorts, with the highest occurrence in the ERCP-naïve group at 36.8%, closely followed by the sphincterotomy-only group at 30.3%. Conversely, the sphincterotomy and stent group demonstrated a starkly lower rate of 7.4% (*p*-value < 0.001). *Klebsiella* spp. was detected in 18.4% of samples, most prevalent in the combined sphincterotomy and stent group at 37.2%, while the sphincterotomy-only and ERCP-naïve groups revealed incidences of 24.2% and 13.0%, respectively (*p*-value < 0.001). The less common *Pseudomonas* spp. (6.7% overall) exhibited a non-significant variance among the cohorts with a *p*-value of 0.080, though the combined intervention group had a slightly elevated rate at 10.6%. Other Gram-negative strains such as *Enterobacter* spp., *Acinetobacter* spp., *and Citrobacter* spp. had their own unique distribution patterns across the patient cohorts, with significant variances observed in *Acinetobacter* spp. and *Citrobacter* spp. at *p*-values of 0.006 and <0.001, respectively.

Regarding the Gram-positive bacteria, *Enterococcus* spp. was observed in 23.6% of all bile samples, markedly concentrated in the sphincterotomy and stent group at a high rate of 43.6%. In comparison, the ERCP-naïve and sphincterotomy-only groups demonstrated incidences of 18.0% and 27.3%, respectively (*p*-value < 0.001). Meanwhile, *Streptococcus* spp. and *Staphylococcus* spp. revealed overall prevalences of 3.9% and 4.1%, respectively. Notably, their distributions across the stratified patient groups were not statistically significant.

Table 6 provides an in-depth analysis into antibiotic resistance patterns among bile cultures differentiated by their respective interventions. In the context of ampicillin/sulbactam, resistance was evident in 33.3% of the samples, sourced from a subset of 66 cases. The previous sphincterotomy group exhibited the lowest resistance rate at 6.1%. In contrast, a slightly elevated resistance was discerned in the group treated with both sphincterotomy and stent placement, at 9.6%. The *p*-value, at 0.036, suggests a statistically significant difference in resistance across these groups.

Piperacillin/tazobactam and fluoroquinolone (ciprofloxacin/levofloxacin) resistance patterns illustrated pronounced disparities with *p*-values of 0.002 and 0.177, respectively. Specifically, the combination of previous sphincterotomy and stent placement reflected a heightened resistance to fluoroquinolones at 19.1%. This suggests that this group may be particularly prone to resist this antibiotic class. Moreover, resistance to penems, encompassing meropenem and imipenem, spanned 17.7% of the samples from 339 cases. The ERCP-naïve group, or the non-intervention group, revealed a resistance rate of 13.0%, in sharp contrast to an absolute non-resistance in the sphincterotomy-only group, implying the potential potency of penems in treating acute cholangitis in patients managed by sphincterotomy.

Delving into resistance patterns concerning different cephalosporin generations, a multifaceted picture emerges. Although resistance to the 2nd and 4th generation cephalosporins did not reveal significant inter-group variations, with *p*-values of 0.441 and 0.246, respectively, the 3rd generation cephalosporin resistance showcased statistically significant differences, with a *p*-value of 0.014. Notably, the sphincterotomy group encountered a heightened resistance rate of 9.1% for these cephalosporins, raising concerns regarding their efficacy for this cohort.

Lastly, the evaluation highlighted pronounced resistance to piperacillin and ticarcillin/clavulanic acid in 55.8% and 32.2% of samples, respectively. These resistance patterns showed substantial variations, reinforced by *p*-values of 0.084 and 0.004. Most strikingly, the combination group of sphincterotomy and stent placement exhibited a marked piperacillin resistance rate of 30.9%.

Table 7 presents an in-depth examination of bile culture outcomes and multidrug resistance patterns between patients with previously placed metal stents and those with plastic stents. For Gram-negative bacteria, *Escherichia coli* was detected in 57.6% of the metal stent group and 52.5% of the plastic stent group (*p* = 0.634). Similarly, *Klebsiella* spp. was found in 57.6% and 44.3% of the metal and plastic stent cohorts, respectively, with an insignificant difference. *Pseudomonas* spp. demonstrated a presence of 18.2% in the metal stent group and 9.8% in the plastic stent group, with a *p*-value of 0.247. *Enterobacter* spp. was less prevalent in both groups, with occurrences of 3.0% and 6.6% for metal and plastic stent patients, respectively (*p* = 0.467). *Acinetobacter* spp. was not detected in either group. *Citrobacter* spp. was found in 15.2% of the metal stent patients and 9.8% of the plastic stent group (with a *p*-value of 0.444).

Regarding Gram-positive bacteria, *Enterococcus* spp. was observed in 36.4% of metal stent patients and 44.3% of plastic stent patients (*p* = 0.458). *Streptococcus* spp. was minimally detected, with a 3.3% occurrence in the metal stent cohort and 4.9% in the plastic stent group, with an insignificant difference in proportions. *Staphylococcus* spp. was involved in 6.1% of the metal stent patients and 1.6% of those with plastic stents (*p*-value = 0.244).

Regarding multidrug resistance, the study observed extended-spectrum beta-lactamases (ESBL) in 21.2% of metal stent patients and 9.8% of plastic stent patients. Methicillin-resistant Staphylococcus aureus (MRSA) was detected in 1.6% of the plastic stent group and was absent in the metal stent group. Vancomycin-resistant enterococci (VRE) were present in 9.1% of the metal stent group, compared to a mere 1.6% in the plastic stent cohort. Carbapenem-resistant Enterobacteriaceae (CRE) were found in 9.1% of metal stent patients and 3.2% of those with plastic stents.

Assessing the bacterial presence in bile samples, a sterile culture was identified in 9.1% of metal stent patients and 3.3% of plastic stent patients. Samples with a single bacterium were noted in 21.2% of the metal stent cohort and 32.8% of the plastic stent group. Bile samples with two bacterial species were identified in 57.6% of metal stent patients and 55.7% of the plastic stent group. Lastly, samples with three or more bacterial species were recorded in 12.1% of metal stent patients and 8.2% of plastic stent patients, without expressing statistical significance.

## 4. Discussion

### 4.1. Literature Findings

In the present study, the primary focus was to delineate the patterns of multidrug resistance and antibiotic resistance in acute cholangitis patients who underwent different intervention strategies such as sphincterotomy, stent placement, or a combination of both. Through the analysis of 488 bile samples, the study identified a substantial presence of multidrug-resistant microorganisms, including ESBL, MRSA, VRE, and CRE, prevalent at varying degrees across different patient groups. Remarkably, the total incidence of multidrug resistance was registered at 19.9%, with a noteworthy proliferation of ESBL, evidenced by a significant variation across groups. The incidence of ESBL was particularly high in the group that had undergone sphincterotomy and had a previous stent at 28.7%. Additionally, Gram-negative *Escherichia coli* exhibited a predominant presence in 30.7% of the total bile samples, with a particularly high prevalence noted in the non-intervention group (43%).

The findings further delineated that the group subjected to both sphincterotomy and prior stent placement exhibited significantly increased susceptibility to multidrug resistance (50%), as well as heightened incidences of VRE (8.5%) and CRE (11.7%), although without statistically significant differences in the case of VRE and CRE. Similarly, a preponderance of Gram-positive *Enterococcus* spp. was detected, specifically in the cohort undergoing both sphincterotomy and previous stent placement, signifying a potential predisposition to Gram-positive bacterial colonization within this group. These data hinted at a complex interplay of interventions and bacterial colonization, suggesting that previous interventions might have influenced the microbial dynamics and resistance patterns in these patients.

Furthermore, the current study provides a comprehensive evaluation of antibiotic resistance patterns, discovering significant divergences, particularly concerning fluoroquinolones and 3rd generation cephalosporins. Resistance to fluoroquinolones was notably heightened in the stent placement group, signifying a considerable vulnerability of this cohort to this category of antibiotics. On the other hand, the resistance to 3rd generation cephalosporins exhibited statistically significant variations, with an increased resistance noticed particularly in the sphincterotomy group, indicating a potential limitation in the efficacy of these antibiotics for this specific group. These findings underscored the necessity to further explore the underlying factors contributing to these divergent resistance patterns, to enhance therapeutic strategies in managing acute cholangitis.

In a retrospective analysis spanning the period from 2010 to 2016, the study by Gromski et al. [33] scrutinized biliary aspirates from more than 700 ERCP procedures in individuals presumed to have acute cholangitis, marking a significant increase of positive samples, compared with a previous study [34]. The study discovered positive bile cultures in about 92% of the analyzed procedures, indicating a predominant consistency in duodenoscope cleaning and culture processing techniques a decade ago. Previous studies suggested a heightened prevalence of certain bacteria including *Enterococcus* spp., *Enterobacter* spp. (23.5% vs. 7.4%), *Pseudomonas. aeruginosa* (12.3% vs. 2.6%), and *Klebsiella.* spp. (53.4% vs. 29.7%) in patients with a biliary stent, a finding not observed in previous research due to a significantly larger sample size and extensive bile culturing in the current study [34,35].

Furthermore, Gromski et al. observed an increase in positive bile culture rates in individuals without prior biliary stent placement, that reached above 85%, in contrast to the 55% recorded in a preceding study, and in our study [33,36]. This surge was hypothesized to be attributed to various factors including modified antibiotic regimens before ERCP or decreased antibiotic resistance in earlier research. Additionally, the investigation noted similar effects of prior biliary endoscopic sphincterotomy on bile culture positivity and microbiology to that of prior biliary stent placement, possibly due to bacterial migration or colonization of the existing biliary stent, supported by the high incidence of biofilm-forming organisms [37,38]. Moreover, a significant portion of multidrug-resistant organisms (23.4%) was identified, with a noted surge in vancomycin-resistant enterococci (VRE) isolation associated with biliary cultures and stents, a correlation previously documented [18], although in our study, VRE was identified in only 2.7% of more than 400 samples. 

In recent years, the medical community has been increasingly concerned with the choice of empiric antibiotics for community-acquired biliary infections. Various societies have suggested the use of third-generation cephalosporins, fluoroquinolones, or penicillin/beta-lactamase inhibitors [39]. Previously, ciprofloxacin was preferred due to a high susceptibility rate in Gram-negative organisms [40]. However, recent data reveal a decline in effectiveness, with susceptibility rates dropping to almost 60% and below 90% for *Escherichia coli* and *Klebsiella* spp., respectively, a trend corroborated globally [37]. This necessitates a reconsideration of initial empiric choices for acute cholangitis, leaning more towards combinations like intravenous fourth-generation cephalosporins and metronidazole [41]. The changes in antibiotic protocol, backed by ongoing studies, reflect a pragmatic approach to tackling increasing antibiotic resistance, while addressing specific regional microbiological trends [42].

Sphincterotomy, a procedure that facilitates bile and pancreatic fluid drainage by incising the sphincter muscle, can potentially introduce bacteria from the intestine into the bile duct. In analyzing antibiotic resistance patterns in acute cholangitis patients, our study revealed that those who underwent sphincterotomy exhibited a unique microbiological profile and had heightened levels of WBC and CRP, indicative of more severe infections. Notably, resistance to piperacillin/tazobactam was pronounced in these patients, suggesting that the procedure can influence microbial dynamics and amplify antibiotic resistance, likely due to recurrent infections and repeated antibiotic use.

In our study, encompassing 488 bile samples, we noted a multidrug resistance rate of 19.9%. The analysis of distinct therapeutic strategies illustrated considerable variations in microbial colonization patterns and resistance based on the type of intervention and antibiotic category. Notably, the group undergoing both sphincterotomy and prior stent placement demonstrated a heightened susceptibility to Gram-positive bacterial colonization, a trend statistically substantiated (*p* < 0.001). The study further revealed that the non-intervention group exhibited the highest prevalence of *Escherichia coli* (43%, *p* < 0.001) and significant resistance variations across different antibiotics, such as piperacillin/tazobactam and fluoroquinolones (*p* = 0.003 and <0.001, respectively). Furthermore, a detailed analysis of stent types revealed significant disparities in bacterial presence and resistance patterns. Metal stent recipients, for instance, were more prone to ESBL infections (18.6%) compared to those with plastic stents (6.2%), and exhibited a greater incidence of polymicrobial infections. These critical data underline the necessity for tailored antibiotic strategies, grounding empirical antibiotic choice in localized study findings to effectively combat the escalating issue of multidrug resistance.

Upon analysis, it can be concluded that the current study findings align well with the trends documented in other significant studies [5,29,43]. These studies also highlighted a predominant presence of *Escherichia coli* in bile cultures in cases of acute cholangitis. Remarkably, our study also noted a similar trend, with a pronounced prevalence of organisms such as *Escherichia coli* in up to 50% of samples, and *Klebsiella* spp. in 20% or more of the obtained samples [44]. Interestingly, unlike several other studies, our research did not detect the presence of anaerobic bacteria such as *Bacteroides fragilis* and *Clostridium* spp., which have been indicated as occasional causative agents in acute cholangitis, especially in patients with a history of biliary operations and the elderly population [45]. This discrepancy could possibly be attributed to the limited sample size, or a high rate of false-negative results commonly associated with anaerobic bacteria.

Additionally, our research validated the suggestions posited by the Tokyo Guidelines concerning the choice of antimicrobial therapy in mild cholangitis. The patterns of resistance to ampicillin/sulbactam observed in our study were aligned with the guidelines, indicating a consistency in the resistance patterns observed across different studies, without considerable differences between benign and malignant causes of cholangitis [46]. This uniformity in data signifies the potential effectiveness of adhering to established guidelines in managing mild cases of cholangitis, and necessitates further research to expand upon these findings and foster optimized therapeutic strategies for acute cholangitis patients.

In the analysis of recurrent cholangitis rates, the researchers found no significant variances between the study groups across all predetermined duration cutoffs. Although our study did not research into antibiotic treatment dose and duration, results from previous studies indicated that the antibiotic treatment length did not significantly influence the recurrence of cholangitis [47,48]. Despite observations of increased bacterial resistance and a higher prevalence of Gram-positive pathogens in recurrent cases [49], the initial empirical treatment schemes seemed inadequate, potentially escalating mortality rates and adverse occurrences in patients experiencing bacteremic acute cholangitis, as mentioned by other studies [50]. In relation to the duration of hospital stays, disparate results were noted across various studies, as well as in our study where patients with malignant etiology of obstruction had significantly longer hospitalization. While some studies [47,51] reported significantly reduced in-hospital durations for patients under short-term treatment, another comprehensive evaluation indicated no notable difference in hospitalization lengths [52]. Despite these discrepancies and high heterogeneity in study data, the potential benefits of shorter antibiotic regimens were affirmed, urging further research to substantiate the possibility of diminished hospital stays, thereby minimizing patient exposure to other complications like thromboembolic events and additional infections [53].

### 4.2. Study Limitations

The study’s retrospective design inherently carries a risk of selection bias and might not account for all confounding factors that could influence the outcomes, compared to a prospective study. The reliance on pre-existing medical records might have introduced information bias, as the completeness and accuracy of data would depend on the quality of the recorded information in the hospital archives and individual patient files. Moreover, the study confined itself to a single tertiary healthcare facility in western Romania, potentially limiting the generalizability of the findings to a wider population, as it might not represent the diverse patient profiles and treatment protocols in other healthcare settings. Also, during the COVID-19 pandemic, shifts in healthcare priorities and the widespread use of antibiotics for secondary infections could have influenced our study’s outcomes on antibiotic resistance in acute cholangitis patients. Patient hesitancy in seeking timely treatment due to pandemic concerns might also have impacted on the observed microbial profiles and severities. Thus, interpreting our findings requires considering this unique context. Lastly, the study focuses extensively on bacterial infections and antibiotic resistance, potentially overlooking other critical biochemical or molecular aspects associated with acute cholangitis, that might predispose to different infection outcomes. 

## 5. Conclusions

This study conclusively underscores the presence of significant variations in multidrug resistance patterns and antibiotic resistance among acute cholangitis patients, primarily influenced by the respective intervention strategies adopted. A noteworthy revelation was the substantial discrepancy in the microbial colonization patterns, heavily dictated by the nature of intervention techniques employed, namely, stent placement and anterior sphincterotomy. Specifically, the study unveiled that the group undergoing both sphincterotomy and previous stent placement showed a predisposition towards ESBL and MDR patterns. Furthermore, a discerning analysis of the antibiotic resistance patterns evidenced considerable fluctuation, hinting at the complex dynamics intrinsic to antibiotic interactions and bacterial resistance to antibiotics. In particular, the elevated resistance to certain antibiotics within specific groups of patients who have previous stent placement or sphincterotomy, accentuates the necessity to re-evaluate and possibly recalibrate the existing antibiotic protocols to counter antibiotic resistance. 

## Figures and Tables

**Table 1:** Background characteristics of patients with acute cholangitis.

Variables	n = 488	%
Age (mean ± SD)	69.3	10.9
Sex	227	46.5%
Male	227	46.5%
Female	261	53.5%
Age category		
Young adults (18–39 years)	21	4.3%
Middle-aged (40–65 years)	138	28.3%
Older adults (>65 years)	329	67.4%
Clinical presentation		
Abdominal pain	358	73.3%
Jaundice	437	89.5%
Fever/Chills	154	31.5%
ERCP timing		
Emergent (<48 h)	318	65.1%
Urgent (48–72 h)	73	14.9%
Late (>72 h)	97	19.9%
Hospitalization days (median, IQR)	8.6	5.3
Weekend admission	132	27.0%
Cholecystectomy	102	20.9%
Tokyo severity score		
Grade I	51	10.5%
Grade II	29	5.9%
Grade III	408	83.6%
Type of intervention		
Previous sphincterotomy	33	6.7%
Previous sphincterotomy and stent	94	19.3%
ERCP-naïve	361	73.8%
Etiology of obstruction		
Malignant	246	50.4%
Benign	242	49.6%

SD—standard deviation; ERCP—endoscopic retrograde cholangiopancreatography; IQR—interquartile range.

**Table 2:** Laboratory data and culture results stratified by intervention history.

Variables	Previous Sphincterotomy (n = 33)	Previous Sphincterotomy and Stent (n = 94)	ERCP-Naïve (n = 361)	*p*-Value
WBC (4.5–11.0 thousands/mm^3^)	11.5 ± 5.8	12.8 ± 6.1	10.6 ± 5.5	0.003
CRP (0–10 mg/L)	108.3 ± 84.0	116.9 ± 96.2	91.5 ± 71.7	0.014
Total bilirubin (0.3–1.2 g/dL)	7.3 ± 6.6	7.8 ± 7.1	6.9 ± 5.2	0.383
PLT (150–450 thousands/mm^3^)	272.5 ± 102.7	266.1 ± 114.9	241.8 ± 96.0	0.041
INR (0.8–1.18)	3.0 ± 1.9	3.3 ± 2.5	1.3 ± 0.4	<0.001
Bile cultures (n = 488)	(n = 33)	(n = 94)	(n = 361)	
Sterile	5 (15.2%)	5 (5.3%)	138 (38.2%)	<0.001
1 bacterium	14 (42.4%)	31 (33.0%)	157 (43.5%)	0.181
2 bacteria	11 (33.3%)	47 (50.0%)	58 (16.1%)	<0.001
≥3 bacteria	3 (9.1%)	11 (11.7%)	8 (2.2%)	0.002
Blood cultures (n = 340)	(n = 18)	(n = 80)	(n = 242)	
Sterile	10 (55.6%)	48 (60.0%)	181 (74.8%)	0.016
1 bacterium	8 (44.4%)	23 (28.8%)	57 (23.6%)	0.119
2 bacteria	0 (0.0%)	9 (11.3%)	4 (1.7%)	0.001
≥3 bacteria	0 (0.0%)	0 (0.0%)	0 (0.0%)	-

WBC—white blood cells; CRP—C-reactive protein; PLT—platelets; INR—international normalized ratio; ERCP—endoscopic retrograde cholangiopancreatography. A *p*-value threshold of less than 0.05 was set for statistical significance.

**Table 3:** Interventions performed based on the etiology of obstruction.

Variables	Previous Sphincterotomy (n = 33)	Previous Sphincterotomy and Stent (n = 94)	ERCP-Naïve (n = 361)	*p*-Value
Benign etiology (n = 242)	(n = 23)	(n = 13)	(n = 206)	
Choledocholithiasis	22 (66.7%)	12 (5.0%)	174 (29.8%)	<0.001
Vaterian ampulloma	0 (0.0%)	0 (0.0%)	4 (1.1%)	0.491
Benign choledochal stenosis	1 (3.0%)	1 (1.1%)	15 (4.2%)	0.342
Mirizzi syndrome	0 (0.0%)	0 (0.0%)	8 (2.2%)	0.239
Liver abscess	0 (0.0%)	0 (0.0%)	5 (1.4%)	0.411
Malignant etiology (n = 246)	(n = 10)	(n = 81)	(n = 155)	
Pancreatic cancer	5 (15.2%)	39 (41.5%)	91 (30.7%)	0.002
Cholangiocarcinoma	5 (15.2%)	23 (9.3%)	22 (6.1%)	<0.001
Malignant vaterian ampulloma	0 (0.0%)	15 (16.0%)	15 (4.2%)	<0.001
Malignant extrinsic compression	0 (0.0%)	4 (4.3.%)	14 (3.9%)	0.501
Gallbladder cancer	0 (0.0%)	0 (0.0%)	3 (0.8%)	0.588

**Table 4:** Evidence of multidrug-resistant microorganisms isolated from bile cultures.

Bile Samples (n = 488)	Previous Sphincterotomy (n = 33)	Previous Sphincterotomy and Stent (n = 94)	ERCP-Naïve (n = 361)	*p*-Value
ESBL = 60 (12.3%)	1 (3.0%)	27 (28.7%)	32 (8.9%)	<0.001
MRSA = 2 (0.4%)	0 (0.0%)	1 (1.1%)	1 (0.3%)	0.528
VRE = 13 (2.7%)	1 (3.0%)	8 (8.5%)	4 (1.1%)	<0.001
CRE = 22 (4.5%)	1 (3.0%)	11 (11.7%)	10 (2.8%)	<0.001
Total MDR = 97 (19.9%)	3 (9.1%)	47 (50.0%)	47 (13.0%)	<0.001

ESBL—extended-spectrum beta-lactamases; MRSA—methicillin-resistant Staphylococcus aureus; VRE—vancomycin-resistant enterococci; CRE—carbapenem-resistant Enterobacteriaceae; MDR—multidrug-resistant.

**Table 5:** Microbial profiles from bile samples by intervention type.

Microbial Identification	Previous Sphincterotomy (n = 33)	Previous Sphincterotomy and Stent (n = 94)	ERCP-Naïve (n = 361)	*p*-Value
Gram-negative				
*Escherichia coli* = 150/488 (30.7%)	10 (30.3%)	7 (7.4%)	133 (36.8%)	<0.001
*Klebsiella* spp. = 90/488 (18.4%)	8 (24.2%)	35 (37.2%)	47 (13.0%)	<0.001
*Pseudomonas* spp. = 33/488 (6.7%)	4 (12.1%)	10 (10.6%)	19 (5.3%)	0.080
*Enterobacter* spp. = 31/488 (6.3%)	2 (6.1%)	5 (5.3%)	24 (6.6%)	0.829
*Acinetobacter* spp. = 10/488 (2.1%)	3 (9.1%)	0 (0.0%)	7 (1.9%)	0.006
*Citrobacter* spp. = 17/488 (3.5%)	2 (6.1%)	10 (10.6%)	5 (1.4%)	<0.001
Gram-positive				
*Enterococcus* spp. = 115/488 (23.6%)	9 (27.3%)	41 (43.6%)	65 (18.0%)	<0.001
*Streptococcus* spp. = 19/488 (3.9%)	2 (6.1%)	3 (3.2%)	14 (3.9%)	0.764
*Staphylococcus* spp. = 20/488 (4.1%)	0 (0.0%)	4 (4.3%)	16 (4.4%)	0.468

**Table 6:** Evaluation of antibiotic resistance patterns from bile cultures stratified by unique cases managed by sphincterotomy, sphincterotomy and stent placement, stent placement, or no intervention.

Antibiotic Resistance	Previous Sphincterotomy (n = 33)	Previous Sphincterotomy and Stent (n = 94)	ERCP-Naïve (n = 361)	*p*-Value
Ampicillin/sulbactam = 22/66 (33.3%)	2 (6.1%)	9 (9.6%)	12 (3.3%)	0.036
Piperacillin/tazobactam = 69/344 (20.1%)	5 (15.2%)	24 (25.5%)	40 (11.1%)	0.002
Fluoroquinolones (ciprofloxacin/levofloxacin = 72/457 (15.8%)	2 (6.1%)	18 (19.1%)	52 (14.4%)	0.177
Penems (meropenem/imipenem) = 60/339 (17.7%)	0 (0.0%)	13 (13.8%)	47 (13.0%)	0.081
2nd gen. cephalosporin = 14/54 (25.9%)	0 (0.0%)	4 (4.3%)	10 (2.8%)	0.441
3rd gen. cephalosporin = 16/356 (4.5%)	3 (9.1%)	6 (6.4%)	7 (2.0%)	0.014
4th gen. cephalosporin = 77/337 (22.8%)	4 (12.1%)	20 (21.3%)	53 (14.7%)	0.246
Aminoglycoside (gentamicin/amikacin) = 14/427 (3.3%)	1 (3.0%)	2 (2.1%)	11 (3.0%)	0.891
Ticarcillin/clavulanic acid = 65/202 (32.2%)	5 (15.2%)	22 (23.4%)	38 (10.5%)	0.004
Piperacillin = 164/294 (55.8%)	6 (18.2%)	29 (30.9%)	131 (36.3%)	0.084

**Table 7:** Bile culture results and multidrug resistance pattern by the type of stent that was previously placed.

Variables	Metal Stent (n = 33)	Plastic Stent (n = 61)	*p*-Value
Gram-negative			
*Escherichia coli* (n = 51)	19 (57.6%)	32 (52.5%)	0.634
*Klebsiella* spp. (n = 46)	19 (57.6%)	27 (44.3%)	0.217
*Pseudomonas* spp. (n = 12)	6 (18.2%)	6 (9.8%)	0.247
*Enterobacter* spp. (n = 5)	1 (3.0%)	4 (6.6%)	0.467
*Acinetobacter* spp. (n = 0)	0 (0.0%)	0 (0.0%)	-
*Citrobacter* spp. (n = 11)	5 (15.2%)	6 (9.8%)	0.444
Gram-positive			
*Enterococcus* spp. (n = 39)	12 (36.4%)	27 (44.3%)	0.458
*Streptococcus* spp. (n = 4)	1 (3.30%)	3 (4.9%)	0.665
*Staphylococcus* spp. (n = 3)	2 (6.1%)	1 (1.6%)	0.244
Multidrug-resistant	(n = 13)	(n = 10)	0.589
ESBL (n = 13)	7 (21.2%)	6 (9.8%)	
MRSA (n = 1)	0 (0.0%)	1 (1.6%)	
VRE (n = 4)	3 (9.1%)	1 (1.6%)	
CRE (n = 5)	3 (9.1%)	2 (3.2%)	
Bacterial presence in bile			0.437
Sterile (n = 5)	3 (9.1%)	2 (3.3%)	
1 bacterium (n = 27)	7 (21.2%)	20 (32.8%)	
2 bacteria (n = 53)	19 (57.6%)	34 (55.7%)	
≥3 bacteria (n = 9)	4 (12.1%)	5 (8.2%)	

ESBL—extended-spectrum beta-lactamases; MRSA—methicillin-resistant Staphylococcus aureus; VRE—vancomycin-resistant enterococci; CRE—carbapenem-resistant Enterobacteriaceae; MDR—multidrug-resistant.

## Data Availability

Data are available on request.
